# Predicting Absolute Risk of Type 2 Diabetes Using Age and Waist Circumference Values in an Aboriginal Australian Community

**DOI:** 10.1371/journal.pone.0123788

**Published:** 2015-04-13

**Authors:** Odewumi Adegbija, Wendy Hoy, Zhiqiang Wang

**Affiliations:** Centre for Chronic Disease, School of Medicine, University of Queensland, Brisbane, Australia; Centre for Cellular and Molecular Biology, INDIA

## Abstract

**Objectives:**

To predict in an Australian Aboriginal community, the 10-year absolute risk of type 2 diabetes associated with waist circumference and age on baseline examination.

**Method:**

A sample of 803 diabetes-free adults (82.3% of the age-eligible population) from baseline data of participants collected from 1992 to 1998 were followed-up for up to 20 years till 2012. The Cox-proportional hazard model was used to estimate the effects of waist circumference and other risk factors, including age, smoking and alcohol consumption status, of males and females on prediction of type 2 diabetes, identified through subsequent hospitalisation data during the follow-up period. The Weibull regression model was used to calculate the absolute risk estimates of type 2 diabetes with waist circumference and age as predictors.

**Results:**

Of 803 participants, 110 were recorded as having developed type 2 diabetes, in subsequent hospitalizations over a follow-up of 12633.4 person-years. Waist circumference was strongly associated with subsequent diagnosis of type 2 diabetes with P<0.0001 for both genders and remained statistically significant after adjusting for confounding factors. Hazard ratios of type 2 diabetes associated with 1 standard deviation increase in waist circumference were 1.7 (95%CI 1.3 to 2.2) for males and 2.1 (95%CI 1.7 to 2.6) for females. At 45 years of age with baseline waist circumference of 100 cm, a male had an absolute diabetic risk of 10.9%, while a female had a 14.3% risk of the disease.

**Conclusions:**

The constructed model predicts the 10-year absolute diabetes risk in an Aboriginal Australian community. It is simple and easily understood and will help identify individuals at risk of diabetes in relation to waist circumference values. Our findings on the relationship between waist circumference and diabetes on gender will be useful for clinical consultation, public health education and establishing WC cut-off points for Aboriginal Australians.

## Introduction

Diabetes is one of the fastest growing chronic conditions in Australia, with an estimated 280 people developing the disease daily [1]. Indigenous Australians (Aboriginals and Torres Strait Islanders) [2, 3] are at higher risk of developing the disease, and at earlier ages [4–6] than the non-indigenous group. A large proportion of Aboriginals develop type 2 diabetes (T2D) in their lifetime, with a lifetime risk in one community of one in two among men and two in three among women [7]. Despite the preventive strategies aimed at controlling the development of type 2 diabetes through healthy diet and lifestyle or medication [8], there has been little, if any reduction in the high prevalence, high burden of mortality and complications imposed by this disease among Aboriginals [1].

Several studies have shown relationship between excess abdominal fat and increased risk of T2D [1, 9, 10]. In Australia, Aboriginals have the propensity for excessive abdominal fat which reflected in their higher waist circumference (WC) compared to non-Aboriginals [11, 12]. Although, studies conducted in some Aboriginal communities have shown WC was a better predictor of T2D compared to body mass index (BMI) and waist-to-hip ratio (WHR) [13, 14], but WC cut-off points for Aboriginals to alert them of the risk of T2D and other chronic diseases have not been established. Prediction models for the risk of diabetes have been developed in a number of populations with the aim of predicting diabetes occurrences, while providing intervention [15–17]. Indeed, the Australian type 2 diabetes risk assessment tool (AUSDRISK) included WC and ethnicity (Aboriginal or non-Aboriginal) as risk factors for estimating the risk of developing diabetes, suggesting the differences in the effects of WC on diabetes between Aboriginals and other Australians [18, 19]. However, due to low numbers of indigenous Australians in the AUSDRISK study, Aboriginals were grouped with southern Europeans and Asians to generate a high-risk group, which does not give a true representation of the level of diabetic risk among Aboriginals in Australia. In this study, we reported the first 10-year absolute risk estimates of diabetes using WC and age in a remote Australian Aboriginal community. As there are no specific WC cut-off thresholds for Aboriginals in Australia, we categorised WC into gender-specific quartiles for analysis and developed a model using WC and age values. To quantify the impact of WC on the risk of developing diabetes, we developed a simplified tool that can be used by health professional and the general public to understand how diabetes risk varies with WC values. This tool can also be used to educate and alert individuals of the risk of developing diabetes according to WC and age. Furthermore, this tool will also be helpful for the planning and conducting obesity-related health education programs for the prevention and management of T2D in Aboriginal communities in Australia.

## Methods

### Study population

The baseline characteristics of the study population have been described in detail elsewhere [13, 20]. From January 1992 to December 1998, a total of 935 adults (> = 18 years, over 80% recruitment) were included in a community-wide screening program in a remote Aboriginal community in Australia’s Northern Territory. Written informed consents were obtained from all participants at baseline measurements. The baseline database (containing screening of anthropometric measurements) was merged with hospitalisation records to identify type 2 diabetes outcomes according to patients’ hospital registration numbers (HRN). Of the 935 individuals, 803 were free of T2D at baseline examination, and were followed up on hospital records for up to 20 years from 1^st^ February 1992 to 31^st^ May 2012. Follow-up stopped for an individual once he/she developed T2D or died. Prior to using the data, each participant was de-identified and given a unique study ID number (SIN). This original baseline database was approved by the Aboriginal community and Ethics Committee of the Menzies School of Health Research and Territory Health Services. The project was approved by the Behavioural and Social Science Ethical Review Committee of the University of Queensland (#2011001232).

### Outcome definition

Participants were followed-up through hospitalization records. Each participant was identified by hospital registration number (HRN) and the patients’ health record ID codes. Our outcome was newly diagnosed (incident) T2D as recorded in hospitalization data records. We identified individuals with T2D using the International Classification of Diseases (9^th^ revision; ICD-9) code 250 and (10^th^ revision, ICD-10) code E11 as recorded in the hospitalization dataset. Follow-up period for participants with incident T2D was the time from the baseline survey date to the diagnosis date. For individuals who did not develop T2D, the follow-up period was the interval between the baseline screening and the follow-up time.

### Waist circumference

Waist circumference (WC) was measured in centimetres (cm) at baseline screening and grouped into gender-specific quartiles for analysis. Quartiles for males: (Q1 = 63–78 cm, Q2 = 79–85 cm, Q3 = 86–95 cm, Q4 = 96–138 cm). Quartiles for females: (Q1 = 60–79 cm, Q2 = 80–90 cm, Q3 = 91–101 cm, Q4 = 101.5–135 cm). Q1 was the reference group for comparison. For the T2D absolute risk prediction, we included WC and age in our model as they have been identified as risk factors for diabetes in Aboriginals [13, 14, 21] and other populations outside Australia [22, 23].

### Statistical methods

Continuous variables were expressed as the mean +/- SD as appropriate. Categorical data were expressed as frequencies and percentages. To assess the association of baseline WC measures on the newly diagnosed T2D, the Cox proportional hazards models were used to estimate hazard ratios (HRs), adjusting for three confounding factors—age, smoking status and alcohol consumption status. Age (years) was included in the analysis as a continuous variable, smoking and alcohol status as categorical variables. The hazard ratios were computed for quartiles Q2, Q3 and Q4 as compared with the lowest quartile (Q1) in different Cox’s proportional hazards regression models. To compare the associations of WC with T2D between males and females, we converted original WC, BMI and WHR values into gender specific z scores for both genders while also controlling for age, smoking and alcohol consumption status. The Weibull regression model was used to predict an individual’s 10-year T2D risk in adult males and females using the formula:

$$Absolute\;risk=1-\exp\left[-{\left\{exp\left(-\beta_{0}-X_{j}\beta_{j}\right)t_{j}\right\}}^{p}\right]$$

where β_0_ represented the baseline WC coefficient, β_j_ was the coefficient for covariates (WC and age), X_j_ represented the covariates, t = time and p = the shape parameter. We constructed the regression coefficient based model by assigning β values as estimated regression coefficients. WC and age were fitted as continuous variables for the estimating the absolute risks of T2D.

For all analyses, two-tailed p values of <0.05 were considered significant. All statistical analyses were performed with STATA version 12.0 [24] and analysis were done separately for males and females. S1 Dataset contains both primary and additional data used for analysis, while S1 Text shows STATA commands used in generating additional data variables used for analysis.

## Results

### Baseline characteristics of study participants

**Fig 1:**
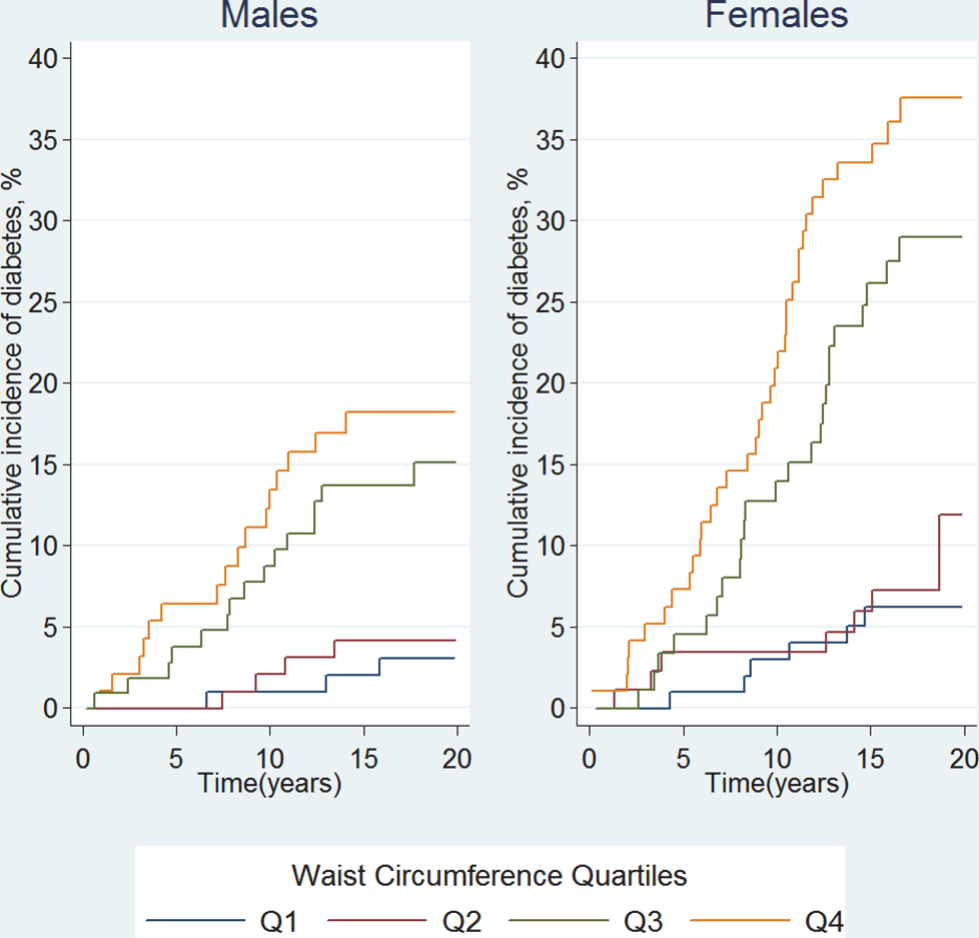
Gender-specific cumulative incidence of diabetes(%) by waist circumference quartiles.

A total of 803 adults who were free from diabetes at baseline records were included in the analysis. The baseline characteristics of participants are shown in **Table 1**. One hundred and ten participants (38 males and 72 females) were diagnosed as having new onset diabetes during the follow-up period of 12633.4 person-years. The median follow-up time was 18 years. The overall diabetes incidence rate was 8.7 (95% CI: 7.2–10.5) per 1,000 person-years, 5.6 (95% CI: 4.1–7.7) per 1,000 person-years for males and 12.2 (95% CI: 9.7–15.4) per 1,000 person-years for females. **Fig 1** shows the cumulative incidence of diabetes (%) according to WC quartiles for males and females respectively in the follow-up time. As WC increased, the cumulative incidence also increased to about 18% for males and 37.5% for females in WC Q4 during the follow-up period.

**Table 1:** Baseline Characteristics of study participants initially free of diabetes in study community followed up for up to 20 years (1992–2012).

	Males	Females
**N**	419	384
**Age (18–76 years)- means (SD)**	32.1 (10.7)	35.0 (12.9)
**Waist circumference (cm)- means (SD)**	85.9 (12.3)	89.0 (14.5)
**Body mass index (kg/m^2^)- means (SD)**	23.0 (4.4)	24.2 (6.0)
**Waist-to-hip ratio- means (SD)**	1.0 (0.1)	0.9 (0.1)
**Smoking status, n (%)**		
**Non-smokers**	76 (18.3)	132 (35.1)
**Smokers**	339 (81.7)	244 (64.9)
**Alcohol consumption status- n (%)**		
**Non-drinkers**	58 (14.0)	237 (63.2)
**Drinkers**	357 (86.0)	138 (36.8)

SD: Standard deviation.

### Hazard ratios from Cox’s proportional hazards regression models

The crude HR for developing T2D with WC as a continuous variable was not significantly different between males and females. Hazard ratio (HR) for 1 cm increase in WC was 1.04 (95%CI: 1.02–1.06, p<0 0001) for males and 1.05 (95%CI: 1.04–1.07, p<0.0001) for females. Crude and adjusted hazard ratios (HR) for WC quartiles are shown in **Table 2**. The observed median follow-up times were 18.6, 18.2, 18.1 and 17.3 years for males and 18.2, 18.1, 16.8 and 15.9 years for females in WC quartiles Q1, Q2, Q3 and Q4 respectively.

**Table 2:** Crude and adjusted hazard ratio and 95% confidence intervals of association between type 2 diabetes and waist circumference quartiles.

WC Quartiles	Crude HR(95%CI)	P-value	*Adjusted HR(95%CI)	P-value
Males				
Q1	1	0.0002	1	0.0003
Q2	1.4 (0.3–6.2)		1.2 (0.3–5.8)	
Q3	4.9 (1.4–17.2)		4.1 (1.2–14.7)	
Q4	6.8 (2.0–23.3)		5.9 (1.6–21.4)	
Females				
Q1	1	<0.0001	1	<0.0001
Q2	1.4 (0.5–4.2)		1.4 (0.5–4.2)	
Q3	5.3 (2.1–12.9)		4.9 (2.0–12.3)	
Q4	7.6 (3.2–18.0)		7.2 (3.0–17.4)	

*Adjusted for age, smoking status and alcohol drinking status.

Waist circumference (WC). Quartile 1 (Q1). Quartile 2 (Q2). Quartile 3 (Q3). Quartile 4 (Q4).

HR (Hazard ratio); 95%CI (95% confidence interval

The HR was 6.8 (95%CI: 2.0–23.4) for males in the highest (Q4) WC quartile compared to those in the lowest (Q1) WC quartile, with p = 0.0002. After adjusting for confounding variables of age, smoking and alcohol status, this association remained statistically significant (HR = 5.9, 95%CI: 1.6–21.4, p = 0.0003). The crude HR for females in Q4 WC quartile was HR = 7.6, (95%CI: 3.2–18.0, p<0.0001). Again, after adjusting for confounding variables, the association remained statistically significant for females, with HR = 7.2 (95%CI: 3.0–17.4, p<0.0001).

**Table 3** shows the crude and adjusted HR of T2D corresponding to 1 standard deviation increase in WC, BMI and WHR (z-scores). The crude hazard ratio for males were 1.7 (95%CI: 1.3–2.2), 1.6 (95%CI: 1.2–2.1) and 1.3 (95%CI: 1.0–1.6) for WC, BMI and WHR respectively. For females, corresponding WC, BMI and WHR crude hazard ratios were 2.1 (95%CI: 1.7–2.6), 1.9 (95%CI: 1.6–2.4) and 1.3 (95%CI: 1.0–1.6). Associations remained statistically significant independently for WC and BMI in both genders after controlling for age, smoking and alcohol consumption. However, the interaction term between gender and WC was not statistically significant (p = 0.22). Likewise, incorporating an interaction term between age and WC was not statistical significant (p = 0.08).

**Table 3:** Crude and adjusted waist circumference, body mass index and waist-to-hip ratio z scores and type 2 diabetes hazards.

	Crude HR(95%CI)	P-value	*Adjusted HR(95%CI)	P-value
**Males**				
Waist circumference (cm)	1.7 (1.3–2.2)	<0.0001	1.7 (1.2–2.3)	0.001
Body mass index (Kg/m^2^)	1.6 (1.2–2.1)	<0.0001	1.6 (1.20 2.2)	<0.0001
Waist-to-hip ratio	1.3 (1.0–1.6)	0.03	1.3 (0.9–1.6)	0.17
**Females**				
Waist circumference (cm)	2.1 (1.7–2.6)	<0.0001	2.0 (1.6–2.5)	<0.0001
Body mass index (Kg/m^2^)	1.9 (1.6–2.4)	<0.0001	1.9 (1.6–2.5)	<0.0001
Waist-to-hip ratio	1.3 (1.0–1.6)	0.05	1.2 (1.0–1.5)	0.10

*Adjusted for age, smoking status and alcohol drinking status.

HR (Hazard ratio); 95%CI (95% confidence interval.

### Absolute risk of diabetes by WC and age

$$Absolute\;risk\;\left(Males\right)=1-\exp\left[-{\left\{exp\left(-8.8008-{\left(-0.0300*WC-0.0353*Age\right)}_{\;}\right)*\;t_{j}\right\}}^{1.1323}\right]$$

$$Absolute\;risk\;\left(Females\right)=1-\exp\left[-{\left\{exp\left(-7.6955-\;{\left(-0.0369*WC-0.0066*Age\right)}_{\;}\right)*\;t_{j}\right\}}^{1.13307}\right]$$

**Fig 2:**
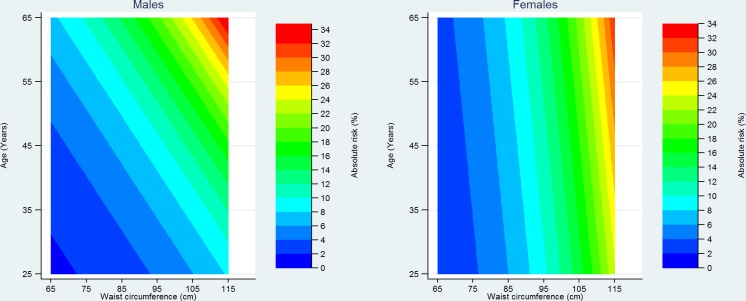
Gender-specific absolute risk(%) of type 2 diabetes, using waist circumference(cm) and age(years).

Based on the coefficients of the final Weibull models above, we estimated 10-year absolute risks according to age and WC values. This was illustrated in **Fig 2,** showing a 10-year absolute diabetes risk (incidence of a first T2D event) of males and females respectively at different WC and age values. Diabetes risk increased with higher WC and older age. For males, the lowest diabetes risk (< = 2%) was for those younger than 30 years with WC less than 73 cm. For females, those with WC less than 75 cm at any age presented the lowest diabetes risk (< = 2%). Absolute diabetes risk of 32–34% was observed in males over 60 years with WC greater than 110 cm and for females over 55 years of age with WC greater than 113 cm.

## Discussion

Using long-term cohort data from a remote community, we have developed a simple model to estimate 10-year risk of T2D in an Aboriginal Australian community based on two variables: WC and age. The results are based on a maximum of 20 years follow-up (median, 18 years) and ascertainment of T2D incidence cases. The values for the two predictors used can easily be obtained by individuals and in clinical practice. The absolute risk chart we developed is simple to use and understand. The availability of the simple tool to predict future risk of T2D should improve the understanding of WC on T2D risk and identify high-risk individuals based on WC and age. It can also serve as an adjunct for planning and conducting public health education programs for T2D and augment preventive strategies for Aboriginal communities.

Our approach based on the absolute risk method calculated from Weibull regression takes into account the synergetic effects of risk factors of disease of interest [25]. This approach took into account the different follow-up time of all participants in the study to predict the risk of the disease. We have presented the prediction to a 10 year period as the goal of this study is to assess how risk of T2D change with WC values with a given age to assist in alerting individuals of the risk of T2D with increasing WC. While T2D is a complex multifactorial disease, and the cause originates from interactions among a number of risk genetic and environmental predictors, increased risk in Australia has been associated with increasing age, family history of diabetes, overweight (particularly with extra weight around the waist region), of indigenous Australian descent, lack of physical activity, unhealthy diet and high blood pressure [26]. Unfortunately, we have no available data for family history of diabetes, physical activity and diet. The focus of this study was to calculate gender-specific absolute risk estimates for a community of Aboriginal Australians according to WC and age while adjusting for age, smoking and drinking in the multivariate analysis. Central obesity measured by WC has been known to increase the risk of T2D in the Australian Aboriginal population [6, 13]. In the present study, we showed WC was a slightly better predictor of T2D than BMI and was much better at predicting T2D than WHR. This is consistent with the study conducted by Wang and Hoy (2004) in the same community presenting the odds ratio for diabetes after adjusting for age and sex to be 2.16, 1.80, 1.41, 1.81 and 1.84 for WC, BMI, weight, WHR, and hip circumference, respectively [13]. As there are no WC cut-off points for Australian Aboriginals, we categorised WC into gender-specific quartiles for the analysis and presented a range for WC (65–115 cm) and age (25–65 years) for the prediction to enable easy use of the information from this study. Age has also been a commonly used single risk factor for detecting undiagnosed diabetes, more importantly, when used in addition to one or more risk factors such as obesity, family history of diabetes and hypertension, has the capacity to identify more individuals with undiagnosed diabetes [27]. An Australian study assessed the relationship of increasing prevalence of diabetes with population ageing and obesity [28]. They considered age group and BMI classification, and found that the greatest relative percentage increases over time were observed among those with normal BMI aged 60 years or older (148%), and those who were obese and less than 60 years of age (139%). While BMI is mostly widely used in Australia, very few studies give reports of WC measured.

A previous Australian diabetes-prediction tool (Australian type 2 diabetes risk assessment tool- AUSDRISK) identified WC, age and ethnicity as risk factors in the prediction of incident diabetes [18, 19]. However, Aboriginals in the AUSDRISK were grouped with Southern Europeans and Asians to generate a high-risk group which does not reflect a true representation of the level of diabetic risk among Aboriginals in Australia. Therefore, the validity and applicability of the tool to Aboriginals in Australia is questionable as they were derived from populations with different risk-factor profiles and ethnicities. The uniqueness of our study lies on our focus on one Aboriginal community; to estimate how the diabetes risk varies according to WC and age values, both have been reported as predictors of diabetes [13, 29].

In our study, the lowest T2D incidence was observed among persons in the lowest WC quartile, and we observed the strongest relationship between WC and T2D in the highest WC quartile. There was statistical significant association between WC and diabetes for both males and females in this population. Our estimates of cumulative incidence in Fig 1 suggest a high incidence of diabetes with high WC particularly among females (37.5%). Based on point estimates, our findings revealed females had higher WC values, and were at higher risk of T2D with increasing WC compared to males. The interaction terms incorporated among the variables used showed no statistical significance. A potential reason for this could be our relatively small sample size resulting in insufficient power to our study in detecting the interactions.

### Strengths and limitations

Our study has a number of strengths. First, to our knowledge, this is the first diabetes prediction model specifically developed for an Aboriginal population in Australia. We focused on Aboriginals in a remote Aboriginal community where they were culturally homogenous (>80% ascertainment of the population). Also focusing on one Aboriginal community minimised the impact of heterogeneity in body habitus, as epidemiological studies on WC in the indigenous population of Australia have shown that there is substantial variation across communities [11]. Second, the prospective study design used minimised systematic error introduced by the recall bias that cross-sectional and case-control studies are subject to. With a follow-up period of up to 20 years with high participation and follow-up rates, there was a robust ascertainment of diabetes events.

A few limitations of our study need to be acknowledged. First, T2D was only ascertained through recording of a T2D diagnosis reported among the diagnoses for a hospitalization episode. It is likely that some people with diabetes were not hospitalised in the community during the study period, so the absolute risk presented in this study could underestimate the true risk in the population. Second, data on other important risk factors of diabetes such as physical activities, diet and family history of diabetes were not available. Therefore, we could only analyse on data available to us (age, WC, smoking, gender, alcohol consumption) in our analysis. Potential confounding effects of other variables (physical activities, family history of diabetes and diet) were not adjusted in the reported strong association between WC and diabetes risk in this study. Third, since our data were from one community, we were unable to generalise our findings about WC-associated absolute risks of T2D in other Australian Aboriginal communities. Due to our relatively small sample size (803 participants and 110 newly developed T2D), we could not assess internal validation by using subset of individuals from the study sample. However, our findings can be further replicated in other Aboriginal communities to assess the generalizability of our findings. Fourth, there may have been inaccuracies of WC measurements at baseline, resulting in the misclassification of participants into WC quartiles and attenuating the observed associations for WC. Finally, the nature of survival analysis with different follow-up time periods for different participants did not permit the calibration of our predicted risks. However, we have shown the gender-specific cumulative incidence for the WC quartiles to observe the proportion of individuals who had diabetes in the follow-up period according to WC quartiles.

## Conclusion

We have constructed a simple tool for predicting the 10- year diabetes risk using WC and age as covariates, and this model focused on Aboriginals in an Australian remote community. This simple tool would assess how absolute risk of T2D changes with WC values. It is also helpful for identifying high-risk individuals, and developing strategies for preventing diabetes in Aboriginal Australians. Our prediction tool will benefit from further validation with the inclusion of other important risk factors such as family history of diabetes, physical activity and diet.

## Supporting Information

S1 Dataset (CSV)

S1 Text STATA commands used for generating secondary data in the dataset. (DOCX)
