# Supplementary material for: Predicting Absolute Risk of Type 2 Diabetes Using Age and Waist Circumference Values in an Aboriginal Australian Community
Source: PLoS One. 2015 Apr 13;10(4):e0123788. doi: 10.1371/journal.pone.0123788 (PMC4395219; doi:10.1371/journal.pone.0123788)
Supplement: S1 Text — (DOCX) [file pone.0123788.s002.docx]

**S2 Text: STATA commands used for generating additional data variables**

* Data for predicting 10-year risk for different age and waist circumference combinations

clear

set obs 2601

egen age1 = seq(), from(20) to(70)

egen waist1 = seq(), from(65) to(115) by(age1)

gen Persontime = 10

gen Diabetes = 0

stset Persontime, failure(Diabetes==1)

save forpredict, replace

* Do-file for generating absolute risk of type 2 diabetes

* Generating model using actual data

import delimited S1 Dataset.csv

* Setting persontime

stset Persontime, failure(Diabetes==1)

* Regression model for absolute risk for males and females separately

foreach s of numlist 1 2 {

streg waist1 age1 if sex1 == `s', d(w) noheader nocnsrep time

preserve

use forpredict, replace

predict S1, s

gen F1=(1-S1)*100

label variable F1 "absolute risk, (%)"

label variable age "Age, (years)"

label variable waist1 "waist circumference, (cm)"

twoway contour F1 age1 waist1 if age>=25 & age<=65, /*

*/ ylabel(25(10)65) xlabel(65(10)120) zlabel(0(2)34, format(%9.0f)) /*

*/ ylab(, angle(0)) ytitle("Age (Years)") /*

*/ xtitle("Waist circumference (cm)") ztitle("Absolute risk (%)") saving(Figure`s'.gph, replace)

graph export Fig`s'.tif, replace

restore

}

exit
